# Developing Machine Learning Algorithms to Support Patient-centered, Value-based Carpal Tunnel Decompression Surgery

**DOI:** 10.1097/GOX.0000000000004279

**Published:** 2022-04-18

**Authors:** Conrad J. Harrison, Luke Geoghegan, Chris J. Sidey-Gibbons, Paul H. C. Stirling, Jane E. McEachan, Jeremy N. Rodrigues

**Affiliations:** From the * Nuffield Department of Orthopaedics, Rheumatology and Musculoskeletal Sciences, University of Oxford, Headington, Oxford, UK; † Section of Vascular Surgery, Department of Surgery and Cancer, Imperial College London, London, UK; ‡ MD Anderson Center for INSPiRED Cancer Care, the University of Texas, Houston, Tex.; § Department of Trauma and Orthopaedic Surgery, Royal Infirmary of Edinburgh, Edinburgh, UK; ¶ Department of Trauma and Orthopaedic Surgery, NHS Fife, Fife, UK; ‖ Warwick Clinical Trials Unit, Warwick Medical School, University of Warwick, Coventry, West Midlands, UK; ** Department of Plastic Surgery, Stoke Mandeville Hospital, Buckinghamshire Healthcare NHS Trust, Ayelsbury, Buckinghamshire, UK.

## Abstract

**Background:**

Carpal tunnel syndrome (CTS) is extremely common and typically treated with carpal tunnel decompression (CTD). Although generally an effective treatment, up to 25% of patients do not experience meaningful benefit. Given the prevalence, this amounts to considerable morbidity and cost without return. Being able to reliably predict which patients would benefit from CTD preoperatively would support more patient-centered and value-based care.

**Methods:**

We used registry data from 1916 consecutive patients undergoing CTD for CTS at a regional hand center between 2010 and 2019. Improvement was defined as change exceeding the respective QuickDASH subscale’s minimal important change estimate. Predictors included a range of clinical, demographic and patient-reported variables. Data were split into training (75%) and test (25%) sets. A range of machine learning algorithms was developed using the training data and evaluated with the test data. We also used a machine learning technique called chi-squared automatic interaction detection to develop flowcharts that could help clinicians and patients to understand the chances of a patient improving with surgery.

**Results:**

The top performing models predicted functional and symptomatic improvement with accuracies of 0.718 (95% confidence interval 0.660, 0.771) and 0.759 (95% confidence interval 0.708, 0.810), respectively. The chi-squared automatic interaction detection flowcharts could provide valuable clinical insights from as little as two preoperative questions.

**Conclusions:**

Patient-reported outcome measures and machine learning can support patient-centered and value-based healthcare. Our algorithms can be used for expectation management and to rationalize treatment risks and costs associated with CTD.

**Takeaways**

**Question:** Can machine learning algorithms be used to predict meaningful improvements in hand function and or symptoms following CTD?

**Findings:** We developed machine learning algorithms using QuickDASH response data from a regional database. The best performing algorithm predicted functional and symptomatic improvement with respective accuracies of 0.72 and 0.76.

**Meaning:** We can identify patients who will benefit from decompression using only two preoperative questions.Our data-driven decision support tools can be used to guide patient selection and both risks and costs of surgery.

## INTRODUCTION

In the United Kingdom, one in 1000 people will undergo carpal tunnel decompression (CTD) to treat carpal tunnel syndrome (CTS) each year.^1^ CTD is generally considered a safe and effective treatment,^1–3^ although 25% of patients do not experience a meaningful improvement in symptoms following the surgery, and 8% deteriorate compared to their preintervention status.^4^ A small number of patients experience significant surgical complications, including median nerve injury,^1^ and this is associated with high litigation costs.^5^

A report commissioned by the UK Department of Health in 2009 suggested that between £0.3 and 0.7 billion could be saved if procedures with “no/limited clinical benefit” were decommissioned.^6^ National Health Service (NHS) England subsequently identified 17 “procedures of limited clinical value,” including CTD, which was recommended only in patients who have evidence of neuropathy, severe symptoms lasting over 3 months or mild-to-moderate symptoms lasting over 4 months after a trial of corticosteroids and/or night splinting.^7^ The evidence base for such strategies is unclear, and they may prove cost ineffective, as the pathway involved involves multiple assessments and treatments. The United States has a similar incidence of CTD to the United Kingdom, although the combined direct and indirect societal cost of the treatment may be even greater, estimated at US $3536.56 ± US $7155.66 per operation.^8^

Previous work has demonstrated large variation in symptom relief following CTD with several factors contributing toward symptomatic improvement following intervention.^9^ Identifying who will derive benefit following CTD will enable the delivery of rational, cost-effective care that optimizes outcomes at the patient-level and reduces unnecessary costs and complications at the population level. If possible, this could be a more effective strategy for the UK NHS, and for the US providers adopting value-based healthcare strategies.^10^

Previous attempts to predict patient-reported outcomes following CTD have failed to capture the different aspects of patient-perceived hand health in detail. Through contemporary psychometrics, it is possible to define success following CTD in terms of either symptomatic or functional improvement, using patient-reported outcome measures (PROMs), factor analysis, and distributional statistics. Machine learning can then be used to model complex statistical relationships between prognostic factors and clinical outcomes.^11^ In this study, we aimed to develop and interpret machine learning algorithms that can predict which patients will demonstrate clinically meaningful improvements in symptoms and/or function following CTD.

## MATERIALS AND METHODS

There are no universally accepted reporting guidelines for development and/or validation studies of machine learning algorithms at present. Therefore, we report this study using relevant items from the Transparent Reporting of a Multivariable Prediction Model for Individual Prognosis or Diagnosis (TRIPOD) checklist,^12^ and from the reporting standards proposed by Luo et al.^13^

### Patient Population

Algorithms were developed as a secondary analysis of data from a clinical practice registry of 1916 consecutive patients undergoing CTD at a regional hand center in Scotland between February 2010 and October 2019. Patients were considered eligible for CTD if they presented with numbness or paresthesia in the distribution of the median nerve with a positive Phalen or Durkan test.^14^ All patients with clinical features suggestive of CTS were then referred for nerve conduction studies. CTD was performed in patients with clinical features of CTS that had not improved following a trial of nonsurgical management for a minimum of 3 months (in the form of steroids and/or night splinting) irrespective of neurophysiological findings. A mini-open approach was used for all patients. Baseline PROM questionnaires were administered on the day of surgery.

### Patient-reported Outcome Measures

We used the QuickDASH to assess changes following surgery. Postoperative QuickDASH scores were taken at least 6 months after surgery (median 12 months). The QuickDASH, consists of 11 items. Each item has five response options, with a higher score indicating poorer upper extremity health. Although designed to have all 11 items contribute to a single summary score, in previous psychometric studies, we have demonstrated that responses to the QuickDASH closely fit a two-factor structural model, in which the task-based QuickDASH items 1–6 measure upper limb function, and QuickDASH items 9–11 measure sensory symptoms. QuickDASH items 7 and 8 relate to social and work activities and do not reliably measure either trait.^15^ This means that more valid measurements can be obtained by splitting the QuickDASH into two subscales, measuring upper limb motor function and sensory symptoms separately.

We used the sum of the item responses to measure function (QuickDASH items 1–6) and symptoms (QuickDASH items 9–11). To determine which patients had experienced a meaningful improvement in either construct following surgery, we calculated the minimal important change (MIC) values for each subscale as half an SD of baseline (preoperative) scores, a widely accepted method.^16^ The MIC represents the minimum change in a patient’s PROM score following intervention that is considered to represent a clinically meaningful improvement.^17^

For both function and symptoms, we performed a missing data analysis and dichotomized patients into those who had and had not improved following surgery (**see Supplemental Digital Content 1**, which displays the methods, appendix, and results, http://links.lww.com/PRSGO/C5). Improvement was defined as a reduction in QuickDASH score greater than the MIC for each subscale. We also used this method to calculate the composite (traditional) QuickDASH MIC. Composite QuickDASH change scores were calculated for each participant using the traditional scoring formula to compare the interpretability of the composite approach to the contemporary two-factor scoring model.

The subscale classifications (improvement versus no improvement in function, and improvement versus no improvement in symptoms) were used for the subsequent machine learning analyses, in which one set of algorithms aimed to predict improvement in symptoms, and another set aimed to predict improvement in function.

### Software

We performed our analyses using the R statistical computing environment (version 4.0.3, **see Supplemental Digital Content 1**, http://links.lww.com/PRSGO/C5). We have made all the code used in this study publicly available for open appraisal and use as a teaching resource.^18^

### Predictors

Our dataset contained 61 predictors (before preprocessing), including demographic and clinical variables, comorbidities, responses to each item in the QuickDASH, Kamath and Stothard^19^ and EuroQol 5-Dimension 5-Level questionnaire (EQ-5D-5L) questionnaires,^20^ baseline symptom and function scores, and whether surgery was to the dominant hand (**see Supplemental Digital Content 1**, http://links.lww.com/PRSGO/C5).

### Algorithms

We trained and tested five different machine learning algorithms that represent a spectrum of model complexity from easily interpretable models with few parameters (eg, regularized logistic regression) to highly complex models (eg, neural networks). As models become more complex, they become more sensitive to subtle relationships within the data, but they also become more likely to find patterns that are not generalizable to the real world, and it becomes less easy to understand how the model has made its predictions. The following models were developed and tested for predicting symptomatic and functional improvement, representing a broad range in algorithmic complexity: a logistic regression with elastic net regularization, a K-nearest neighbors algorithm, a support vector machine, a random forest built through extreme gradient boosting (XGB), and an artificial neural network.

### Model Training and Testing

For each set of algorithms, the data were randomly split into training (75%) and test (25%) sets. The models taught to find patterns in the training dataset, and the evaluated in the test dataset (**see Supplemental Digital Content 1**, http://links.lww.com/PRSGO/C5). Models were compared by classification accuracy, area under the receiver operating characteristic curve (AUC), sensitivity and specificity. We estimated 95% confidence intervals (CIs) for these metrics through nonparametric bootstrapping.

### Model Explanations

For each classification task, we identified the best performing model based on test data classification accuracy. To understand how these models were making their predictions, we performed Shapley additive explanations (SHAP) on their test dataset predictions.^21^ This estimates the contribution of each predictor to the overall model prediction by rerunning the algorithm on many artificially altered versions of the dataset, then measuring how a specific predictor’s presence or value affects the overall prediction. Although this technique cannot unequivocally demonstrate a complex model’s inner workings, it can provide insights into patterns within the training data, and some assurance that the model is making rational predictions based on plausible data patterns.

### Heuristic Models

Finally, we aimed to develop simple, heuristic models (decision trees) that could guide the selection of patients for CTD without the need for sophisticated technology. To do this, we used chi-squared automatic interaction detection (CHAID) to create decision trees that grouped patients into those who were more or less likely to benefit from surgery, based on their response to one or two preoperative questions. This is innovative in clinical research and allows the five-level polytomous (multiple-choice) PROM responses to be handled, rather than collapsed into dichotomous groups, which is needed for other techniques.^22^

We created one decision tree to predict functional improvement, and a second to predict symptomatic improvement. In CHAID, each variable is treated as categorical, we therefore categorized EQ-5D visual analog scale (VAS) scores as less than 70, 70–79, 80–89, or greater than 89, based on the score distributions in our dataset. QuickDASH item response scores were treated as ordinal data.

## RESULTS

### Demographics

Our dataset included item responses and outcome measurements from 1916 patients. For models that predicted symptomatic improvement following CTD, we included 1093 of 1916 patients who had complete response sets to QuickDASH items 9–11 preoperatively and postoperatively. Of the 823 patients with incomplete response sets, 792 were missing postoperative item responses. For models that predicted functional improvement following CTD, we included 1045 of 1916 patients who had complete response sets to QuickDASH items 1–6 preoperatively and postoperatively. Of the 871 patients with incomplete response sets, 839 were missing postoperative item responses. A missing data analysis suggested that missing postoperative responses were largely missing at random^23^ (**see Supplemental Digital Content 1**, http://links.lww.com/PRSGO/C5). Table 1 provides demographics and clinical details for the 1045 patients in whom we could measure functional change and 1093 patients in whom we could measure symptomatic change.

**Table 1.** Patient Demographics and Clinical Details

		Function Models	Symptoms Models
Age, y		61 (22)	62 (22)
Gender	Female	686 (65.6%)	714 (65.3%)
	Male	356 (34.1%)	376 (34.4%)
Hand dominance	Left	104 (10.0%)	107 (9.8%)
	Right	929 (88.8%)	972 (88.9%)
Undergoing surgery to dominant hand	No	420 (40.2%)	430 (39.3%)
	Yes	593 (56.7%)	630 (57.6%)
Diagnosis	CTS	1032 (98.8%)	1078 (98.6%)
	Recurrent CTS	13 (1.2%)	15 (1.4%)
Symptom duration (mo)		20 (24)	20 (24)
Preoperative splinting time (mo)		6 (9)	6 (9)
Smoking status	Nonsmoker	899 (86.0%)	940 (86.0%)
	Smoker	136 (13.0%)	144 (13.2%)
Heart disease	No	912 (87.3%)	944 (86.4%)
	Yes	126 (12.1%)	143 (13.1%)
High blood pressure	No	618 (59.1%)	638 (58.4%)
	Yes	421 (40.3%)	450 (41.2%)
Lung disease	No	964 (92.2%)	997 (91.2%)
	Yes	72 (6.9%)	85 (7.8%)
Diabetes	No	893 (85.5%)	932 (85.3%)
	Yes	148 (14.2%)	157 (14.4%)
Stomach ulcers	No	985 (94.3%)	1025 (93.8%)
	Yes	53 (5.1%)	57 (5.2%)
Kidney disease	No	1005 (96.2%)	1050 (96.1%)
	Yes	31 (3.0%)	34 (3.1%)
Liver disease	No	1026 (98.2%)	1071 (98.0%)
	Yes	13 (1.2%)	15 (1.4%)
Anemia	No	1000 (95.7%)	1050 (96.1%)
	Yes	34 (3.3%)	31 (2.8%)
Cancer	No	985 (94.3%)	1029 (94.1%)
	Yes	50 (4.8%)	52 (4.8%)
Depression	No	879 (84.1%)	919 (84.1%)
	Yes	152 (14.5%)	157 (14.4%)
Osteoarthritis	No	697 (66.7%)	725 (66.3%)
	Yes	334 (32.0%)	351 (32.1%)
Back pain	No	669 (64.0%)	705 (64.5%)
	Yes	371 (35.5%)	379 (34.7%)
Rheumatoid arthritis	No	960 (91.9%)	1007 (92.1%)
	Yes	64 (6.1%)	64 (5.9%)
Thyroid disease	No	902 (86.3%)	940 (86.0%)
	Yes	124 (11.9%)	133 (10.3%)
Preoperative EQ-5D VAS		80 (20)	80 (20)
Follow-up time (mo)		12 (0)	12 (0)
Baseline symptoms score (possible range 3–15)		10 (3)	9 (3)
Baseline function score (possible range 6–30)		16 (9)	16 (3)
Improvement in symptoms	No	240 (23.9%)	267 (24.4%)
	Yes	764 (76.1%)	826 (75.6%)
Improvement in function	No	538 (51.5%)	517 (51.5%)
	Yes	507 (48.5%)	487 (48.5%)

Continuous variables are presented as median (interquartile range) and categorical variables are presented as totals (percentage of the total number of participants in the respective group, including those with missing data). Comorbidities were all self-reported. Improvement is defined as a drop in score greater than the minimal important change estimate.

### Minimal Important Change

There were 1889 preoperative response sets that contained at least ten item responses, which is a prerequisite for calculating QuickDASH scores using the traditional scoring formula. Using this formula, the SD of baseline scores was 20.7, resulting in a half SD MIC estimate of 10 QuickDASH points. Of these 1889 patients, 1117 had at least ten postoperative item responses and 705 reported a drop in score of more than 10 QuickDASH points. In other words, according to the traditional QuickDASH scoring, 63% of participants experienced a meaningful improvement in upper extremity health following surgery.

This was compared to an approach that accounted for the two-factor structure of the QuickDASH. There were 1851 preoperative response sets that contained responses to the function items 1–6, and 1861 that contained responses to the symptom items 9–11. The SD of the function item sum scores (which could range from 6 to 30) was 6.1. The SD of the symptom item sum scores (which could range from 3 to 15) was 2.4. Consequently, the 0.5 SD MIC estimates for the function and symptom subscales were three and two points, respectively. Complete preoperative and postoperative function item response sets were available for 1045 patients and complete preoperative and postoperative symptom item response sets were available for 1093 patients. Using a change larger than the function- and symptom-specific MIC estimates to define meaningful improvement, 507 (49%) patients reported a meaningful improvement in function following surgery and 826 (76%) reported a meaningful improvement in symptoms. For each subscale, all nonresponders had a baseline score higher than the respective MIC, and so had the potential to improve by at least the MIC in that subscale.

### Model Performance

The performance of each trained model when applied to the test dataset is presented in Table 2. The best performing model for predicting meaningful functional improvement was the XGB, with an accuracy of 0.718 (95% CI 0.656, 0.711). The best performing model for predicting meaningful symptomatic improvement was also an XGB, with an accuracy of 0.766 (95% CI 0.719, 0.814). Full confusion matrices for each model are available in the **Results section**, **Supplemental Digital Content 1** (http://links.lww.com/PRSGO/C5).

**Table 2.** Performance of Models Trained to Predict Functional and Symptomatic Improvement Exceeding the Minimal Important Change

Models Trained to Predict Meaningful Functional Improvement				
	Accuracy	AUC	Sensitivity	Specificity
EN	0.698 [0.638, 0.752]	0.779 [0.719, 0.831]	0.659 [0.575, 0.736]	0.737 [0.659, 0.810]
KNN	0.679 [0.622, 0.737]	0.741 [0.680, 0.802]	0.705 [0.626, 0.789]	0.654 [0.568, 0.733]
SVM	0.706 [0.649, 0.760]	0.786 [0.725, 0.840]	0.674 [0.592, 0.754]	0.737 [0.656, 0.809]
XGB	0.718 [0.660, 0.771]	0.791 [0.731, 0.844]	0.736 [0.653, 0.810]	0.699 [0.622, 0.772]
ANN	0.660 [0.599, 0.718]	0.714 [0.651, 0.772]	0.659 [0.580, 0.742]	0.662 [0.583, 0.740]
**Models trained to predict symptomatic improvement**				
	**Accuracy**	**AUC**	**Sensitivity**	**Specificity**
EN	0.708 [0.653, 0.763]	0.749 [0.685, 0.812]	0.755 [0.695, 0.811]	0.571 [0.461, 0.687]
KNN	0.613 [0.555, 0.668]	0.591 [0.513, 0.663]	0.676 [0.555, 0.668]	0.429 [0.309, 0.543]
SVM	0.661 [0.602, 0.712]	0.669 [0.590, 0.739]	0.696 [0.632, 0.755]	0.557 [0.443, 0.662]
XGB	0.759 [0.708, 0.810]	0.733 [0.663, 0.804]	0.868 [0.819, 0.913]	0.443 [0.333, 0.574]
ANN	0.668 [0.610, 0.723]	0.655 [0.585, 0.723]	0.750 [0.694, 0.807]	0.429 [0.313, 0.548]

Figures are presented as: statistic [95% confidence interval].

ANN, artificial neural network; EN, logistic regression with elastic net regularization; KNN, K-nearest neighbors; SVM, support vector machine.

**Fig. 1.**
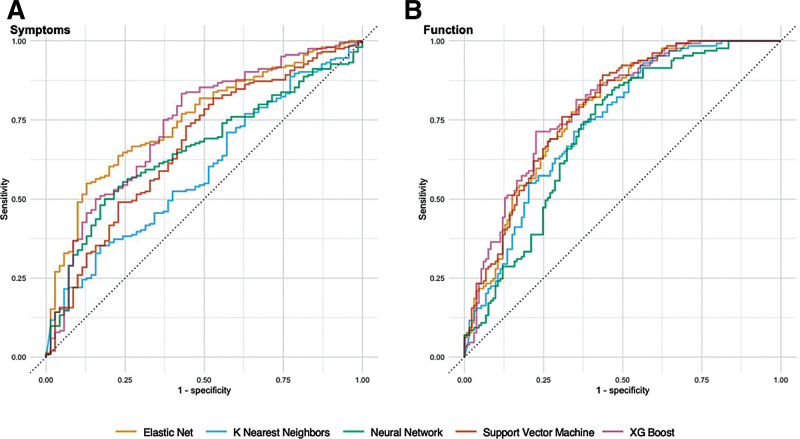
Receiver operating characteristic curves for each classifier algorithm. A, The symptomatic improvement classifiers. B, The functional improvement classifiers.

Figure 1 demonstrates receiver operating characteristic (ROC) curves for each model when applied to the test data.

### Model Explanations

For the functional improvement classifier, the two most important predictors of improvement were baseline function and the ability to use a knife to cut food, reflected in QuickDASH item 5. For the symptomatic improvement classifier, the two most important predictors of improvement were QuickDASH item 10 and baseline mobility, as captured by the EQ-5D mobility domain. Shapley values for all features are presented in the **Results section**, **Supplemental Digital Content 1** (http://links.lww.com/PRSGO/C5).

### Heuristic Models

**Fig. 2.**
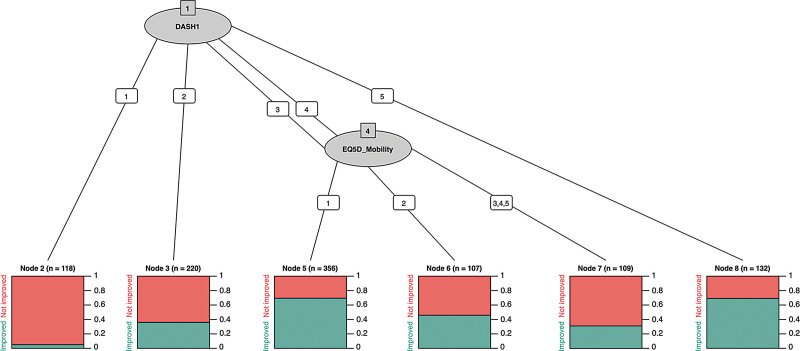
Decision tree for predicting functional improvement following CTD, based on CHAID. Ovals represent PROM items and numbered lines indicate the response to each item. In this case, a response of 3 or 4 to QuickDASH item 1 would trigger the administration of the EQ-5D-5L mobility domain. Underlying bar charts demonstrate the proportion of patients in each node that experienced meaningful improvement in function (green) and no meaningful improvement in function (red).

**Fig. 3.**
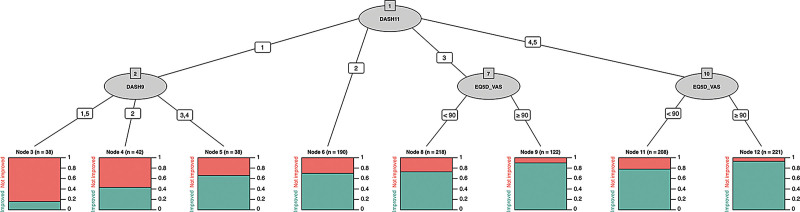
Decision tree for predicting symptomatic improvement following CTD, based on CHAID. Ovals represent PROM items and numbered lines indicate the response to each item. In this case, a response of 4 or 5 to QuickDASH item 11 would trigger the administration of the EQ-5D-5L visual analog scale. Underlying bar charts demonstrate the proportion of patients in each node that experienced meaningful improvement in symptoms (green) and no meaningful improvement in function (red).

Our CHAID trees are demonstrated in Figures 2 and 3. Overall, CHAID suggested that better overall baseline health and more severe hand symptoms made patients more appealing candidates for surgery. Within our dataset, 96% of 118 patients that reported no difficulty opening a jar (response 1 to QuickDASH item 1) experienced no functional improvement following surgery (Fig. 2). The higher the response to this item, the greater the frequency of improvement. Additionally, the lower response to the EQ-5D-5L mobility domain, the greater the frequency of improvement. In other words, patients that had more difficulty opening a jar, and better overall mobility were more likely to experience a meaningful functional improvement following CTD within our cohort.

We found similar results with the symptomatic improvement decision tree. Within our registry, 93% of respondents who reported pain causing severe difficulty in sleeping, or, an inability to sleep (responses 4 or 5 to QuickDASH item 11), but were otherwise in good health (an EQ-5D-5L VAS score of 80 or above), experienced a meaningful improvement in symptoms following surgery.

Those experiencing extreme pain (response option 5 to QuickDASH item 9), but no difficulty sleeping due to the pain (response 1 to QuickDASH item 11) were unlikely to improve, with a 16% improvement rate within this group.

## DISCUSSION

We have developed algorithms that can predict clinically meaningful improvement in either hand function or symptoms following CTD. We have quantified the effect of individual predictors on overall prediction accuracy and have developed heuristic decision support tools that can be readily implemented in clinical practice to identify patients who are likely or unlikely to benefit from CTD, using only two preoperative questions. This could take the form of wall-mounted flow diagrams with one or two steps, as per our CHAID trees in Figures 2 and 3.

Given the perceived low risk of the procedure, and the absence of a consensus gold standard for diagnosing CTS, CTDs may be performed with a shared acceptance of some diagnostic uncertainty. However, in our registry, all procedures were conducted with therapeutic (and not diagnostic) intent. Every patient was deemed likely to improve by at least one experienced hand surgeon, based on clinical assessment and more ready access to neurophysiology investigations than is typically available. Our models were able to detect patients in this cohort that would not improve following surgery, and these patients had not been ruled out by expert surgeons alone. Our findings suggest that, if used to support shared decision-making, expert surgical opinion combined with these models may help to further reduce the costs and risks associated with unbeneficial surgery, as well as supporting expectation management. In the financial year 2018/2019 (before the COVID-19 pandemic) 44,540 CTDs were performed in the NHS.^24^ Assuming 25% (11,135 patients) experience no improvement following CTD, the NHS will spend more than £12 million per year on CTD in patients that experience no discernible benefit (based on 2021/2022 tariff of £1120 for CTD).^25^ On top of these potential direct cost savings, clinical application our algorithm could also lead to indirect cost savings through societal and litigation costs associated with unnecessary intervention.

Also, we have demonstrated that the interpretability of the QuickDASH is improved by separating function items from symptom items. Conventional scoring of all QuickDASH items on an ordinal scale may mean that clinically relevant changes in upper extremity health are not detected. When intervention effect was quantified using traditional composite scoring, 63% of patients in our study experienced a clinically meaningful improvement in upper extremity health. However, when the two-factor structure of the QuickDASH was considered, 49% of patients experienced improvement in upper extremity function and 76% of patients experienced a clinically meaningful improvement in symptoms following CTD. The latter is consistent with the reported success rate of CTD when quantified using other patient-reported measures.^4,26^ The clinical value of CTD may be underestimated when traditional scoring of the QuickDASH is used, as improvements in symptoms may be diluted by static overall function. The latter being affected by other issues such as general physical state and comorbidities. Future CTD research involving the QuickDASH might involve cognitive debriefing through qualitative work, to confirm whether “symptom” scoring is what matters to patients. If so, this would support factor-based scoring being used in future studies, and refine our understanding of hand status measurement in general.

We have demonstrated that, in this dataset, baseline function is the most important predictor of functional improvement following CTD. Furthermore, the two most important predictors of symptomatic improvement were QuickDASH item 10 (corresponding to paresthesia severity) and baseline mobility (EQ-5D Mobility Domain score). Our finding that higher symptom severity is associated with a greater likelihood of symptomatic improvement is in contrast with Jerosch-Herold et al.,^27^ who report lower symptom severity as an independent prognostic factor in the prediction of a positive outcome following CTD (quantified using CTS-6 score using multiple univariate logistic regression).

There is evidence that baseline patient-reported outcomes play a substantial role in the prediction of postoperative outcomes. Pfob et al.^28^ demonstrate that high baseline breast satisfaction is a principal determinant of poor patient satisfaction following breast reconstruction (quantified via the BREAST-Q PROM). The relationship between baseline PROs and postoperative outcomes are further convoluted in the context of CTS as determinants of baseline health may be distinct from determinants of baseline upper extremity health. Our finding that patients with limited upper extremity function but better global mobility were more likely to experience a clinically meaningful improvement following CTD, is in line with Jerosch-Herold et al.^27^ who identified higher baseline health (quantified using EQ-5D-3L utility values) as an independent prognostic factor of positive outcome following CTD using a global rating of change scale.

Our study has limitations. We used data from a large single-center database with procedures performed by a small number of expert surgeons. This potentially limits the generalizability of our findings. Future external and prospective validation is required to assess the effectiveness of our tools as clinical decision support systems in other populations. Dichotomizing outcomes as either improved or not improved may represent an oversimplification of the response to intervention, though we took steps to explore this, such as confirming that many nonresponders had the potential to achieve benefit. Furthermore, CTD may prevent progression of symptoms rather than alleviate them or improve hand function, meaning patients remain at baseline and thus do not improve. The temporal relationship between baseline symptoms, surgery, and response cannot be quantified outside of a prospective trial with a nonoperative comparator.

Our work demonstrates the future synergistic potential of routine PROM collection, contemporary psychometrics, and applied machine learning in the development of data-driven decision support systems. Such systems have already emerged in clinical practice to support decisions regarding patient suitability for hip and knee arthroplasty and hold promise in the streamlining of care pathways from initial presentation in primary care through to definitive intervention.^29^

At present, there exist a number of logistical, sociocultural and ethical barriers to the implementation of machine learning algorithms in clinical practice.^30^ To address these barriers, we have developed heuristic decision trees that can be readily integrated into existing clinical decision-making systems and immediately impact patient care.

## CONCLUSIONS

The value of CTD must be determined using interpretable outcome measures on a patient-specific basis. This can be achieved with the QuickDASH PROM, following a two-factor structure. Machine learning algorithms and heuristic decision support tools can then predict functional and symptomatic improvement following CTD. In the near future, decision support tools could leverage the predictive power of PROMs and machine learning to support patient-centered decision support in hand surgery clinics.

## Supplementary Material


