# Supplementary material for: Developing Machine Learning Algorithms to Support Patient-centered, Value-based Carpal Tunnel Decompression Surgery
Source: Plast Reconstr Surg Glob Open. 2022 Apr 18;10(4):e4279. doi: 10.1097/GOX.0000000000004279 (PMC9015194; doi:10.1097/GOX.0000000000004279)
Supplement: Supplementary file 1 [file gox-10-e4279-s001.pdf]

Supplementary Appendix: Supplementary Methods

Software, Hardware and Code Availability

All analyses were performed in R version 4.0.3 with the following packages: *dplyr* v 1.0.6, *finalfit* v 1.0.2, *performanceEstimation* v 1.1.0, *recipes* v 0.1.16, *keras* v 2.4.0, *parsnip* v 0.1.5, *kernlab* v 0.9-29, *dials* v 0.0.9, *ggplot2* v 3.3.3, *rsample* v 0.0.9, *tune* v 0.1.3, *workflows* v 0.2.2, *yardstick* v 0.0.7, *xgboost* v 1.4.1.1, *SHAPforxgboost* v 0.1.1, *kknn* v 1.3.1, *CHAID* v 0.1-2.

All analyses were performed on a 2017 MacBook Air running macOS Mojave version 10.14.6 on a 1.8GHz Intel Core i5 processor with 8 GB of random-access memory (RAM).

We used the *tidymodels* architecture to train and test our machine learning algorithms. We have made all our code publicly available on the first author's GitHub page: https://github.com/MrConradHarrison/ML-for-Carpal-Tunnel.

Missing Data

To develop machine learning algorithms that predicted symptomatic improvement following CTD, we included 1093/1916 patients who had complete response sets to QuickDASH items 9-11 preoperatively and postoperatively. Of the 823 patients with incomplete response sets, 792 were missing postoperative item responses.

To develop machine learning algorithms that predicted functional improvement following CTD, we included 1045/1916 patients who had complete response sets to QuickDASH items 1-6 preoperatively and postoperatively. Of the 871 patients with incomplete response sets, 839 were missing postoperative item responses.

Predictors of missing postoperative responses were identified through the *finalfit* package (see our R script entitled "02 Missing data analysis.R")

Features

Supplementary Table 1 describes the featrues included in our dataset prior to preprocessing.

**Feature code**	**Description**
Age	Patient age in years (continuous integer)
Gender	Patient gender (categorical: female or male)
Pregnancy	Pregnancy status at time of operation (categorical: no or yes)
HandDominance	Patient hand dominance (categorical: left or right)
LengthSymptoms	Duration of symptoms in months (continuous integer)
Previous_injections	Number of previous steroid injections into the affected carpal tunnel (continuous integer)
Previous_Operations	Number of previous operations on the affected carpal tunnel (continuous integer)
VibratingToolsYN	Whether the patient works with vibrating tools (categorical: no or yes)
VibrationPerWeek	The number of hours per week that the patient works with vibrating tools (continuous integer)
VibrationYears	The number of years for which the patient has worked with vibrating tools (continuous integer)
SplintMonths	The number of months in which the patient underwent preoperative splinting (continuous integer)
SmokingStatus	The patient's current smoking status (categorical: Non_smoker or Smoker)
CigarettePerDay	The number of cigarettes the patient smokes a day (continuous integer)
CigaretteYears	The number of years for which the patient has smoked (continuous integer)
heart	Self-reported current heart disease, treated or untreated (categorical: no or yes)
hbp	Self-reported current high blood pressure, treated or untreated (categorical: no or yes)

lung	Self-reported current lung disease, treated or untreated (categorical: no or yes)
diabetes	Self-reported current diabetes, treated or untreated (categorical: no or yes)
ulcer	Self-reported current stomach ulcers, treated or untreated (categorical: no or yes)
kidney	Self-reported current kidney disease, treated or untreated (categorical: no or yes)
liver	Self-reported current liver disease, treated or untreated (categorical: no or yes)
anaemia	Self-reported current anaemia, treated or untreated (categorical: no or yes)
cancer	Self-reported current cancer, treated or untreated (categorical: no or yes)
depression	Self-reported current depression, treated or untreated (categorical: no or yes)
osteoarthritis	Self-reported current osteoarthritis, treated or untreated (categorical: no or yes)
backpain	Self-reported current back pain, treated or untreated (categorical: no or yes)
rheumatoid	Self-reported current rheumatoid arthritis, treated or untreated (categorical: no or yes)
Thyroid	Self-reported current thyroid disease, treated or untreated (categorical: no or yes)
EQ5D_Mobility	Response to the EQ-5D-5L Mobility Dimension (continuous integer: 1-5)
EQ5D_Selfcare	Response to the EQ-5D-5L Self Care Dimension (continuous integer: 1-5)
EQ5D_Usual_Activities	Response to the EQ-5D-5L Usual Activities Dimension (continuous integer: 1-5)
EQ5D_Pain	Response to the EQ-5D-5L Pain/Discomfort Dimension (continuous integer: 1-5)
EQ5D_Anxiety	Response to the EQ-5D-5L Anxiety/Depression Dimension (continuous integer: 1-5)
EQ5D_VAS	Response to the EQ-5D-5L visual analogue scale (continuous integer: 1-100)
DASH1	Response to QuickDASH item 1, relating to difficulty in opening a tight jar (continuous integer: 1-5)
DASH2	Response to QuickDASH item 2, relating to difficulty doing household tasks (continuous integer: 1-5)
DASH3	Response to QuickDASH item 3, relating to difficulty carrying shopping (continuous integer: 1-5)
DASH4	Response to QuickDASH item 4, relating to difficulty washing one’s back (continuous integer: 1-5)
DASH5	Response to QuickDASH item 5, relating to difficulty in using a knife (continuous integer: 1-5)
DASH6	Response to QuickDASH item 6, relating to difficulty in forceful recreational activities (continuous integer: 1-5)
DASH7	Response to QuickDASH item 7, relating to interference with social activities (continuous integer: 1-5)
DASH8	Response to QuickDASH item 8, relating to interference with work activities (continuous integer: 1-5)
DASH9	Response to QuickDASH item 9, relating to pain severity (continuous integer: 1-5)
DASH10	Response to QuickDASH item 10, relating to paresthesia severity (continuous integer: 1-5)
DASH11	Response to QuickDASH item 10, relating to night pain (continuous integer: 1-5)
KS1	Response to item 1 on the Kamath and Stothard Carpal Tunnel Questionnaire, relating to night pain (categorical: no or yes)
KS2	Response to item 2 on the Kamath and Stothard Carpal Tunnel Questionnaire, relating to paresthesia and hypoesthesia at night (categorical: no or yes)
KS3	Response to item 3 on the Kamath and Stothard Carpal Tunnel Questionnaire, relating to the relative severity of paresthesia and hypoesthesia in the morning (categorical: no or yes)

KS4	Response to item 4 on the Kamath and Stothard Carpal Tunnel Questionnaire, relating to hand or wrist movements that relieve symptoms (categorical: no or yes)
KS5	Response to item 5 on the Kamath and Stothard Carpal Tunnel Questionnaire, relating to paresthesia or hypoesthesia in the little finger (categorical: no or yes)
KS6	Response to item 6 on the Kamath and Stothard Carpal Tunnel Questionnaire, relating to paresthesia or hypoesthesia while reading a newspaper, driving or knitting (categorical: no or yes)
KS7	Response to item 7 on the Kamath and Stothard Carpal Tunnel Questionnaire, relating to neck pain (categorical: no or yes)
KS8	Response to item 8 on the Kamath and Stothard Carpal Tunnel Questionnaire, relating to severity of paresthesia or hypoesthesia during pregnancy (categorical: no or yes)
KS9	Response to item 9 on the Kamath and Stothard Carpal Tunnel Questionnaire, relating to the effectiveness of splinting for symptom relief (categorical: no or yes)
Employment_Status	Patient's current employment (categorical: Worker, Unemployed or Long Term Sick / Disabled.
BMI	Patient's BMI at time of surgery (continuous integer)
Diagnosis	Patient's diagnosis (categorical: CTS or Recurrent CTS)
laterality	Laterality of surgery (categorical: unilateral or bilateral)
DominantSideSurgery	Whether carpal tunnel decompression was performed on the patient's dominant hand (categorical: no or yes)
Baseline_symptoms	The sum of preoperative item responses to QuickDASH items 9-11
Baseline_function	The sum of preoperatice item responses to QuickDASH items 1-6

Supplementary Table 1. Feature codes and descriptions of the features included in our dataset (prior to preprocessing).

Preprocessing

For both the symptomatic improvement and functional improvement classifiers, training dataset class balance (improved vs not improved) was checked prior to bootstrapping and hyperparameter tuning. Class imbalance in the symptomatic improvement classifier training data was addressed with a synthetic minority oversampling technique (SMOTE) algorithm.[1]

Dummy variables were created for categorical predictors via level encoding. Continuous predictors were scaled by 2 standard deviations. Missing data were imputed via mode imputation for categorical predictors and mean imputation for continuous predictors. Predictors with a near zero variance were removed (see scripts "04 Symptom classification.R" and "05 Function classification.R")

Hyperparameter Tuning

Model hyperparameters were tuned using grid search implemented through the *tidymodels* framework.[2] Regular grids with 10 levels per tuned hyperparameter were used for this process, except for the extreme gradient boosted decision tree ensemble (XGB), which has a large parameter space. In this case, Latin hypercube sampling was used to generate the hyperparameter grid.[3]

For the logistic regression with elastic net regularization, penalty and mixture hyperparameters were tuned, relating to the shrinkage operator and the proportion of L1:L2 regularization respectively.

For the XGB, the number of trees was set to 1000, while we tuned: tree depth, the minimum number of observations per node required to undertake a split, the loss reduction required to undertake a split, the sample size, the number of predictors sampled per split and the learn rate.

For the support vector machine (SVM) the cost penalty and polynomial degree were tuned. For the ANN, the number of hidden layers and dropout rate were tuned over 10 epochs. For the K nearest neighbors (KNN) algorithm, the number of neighbors and choice of kernel function were tuned.

Optimal model hyperparameters were selected from the bootstrapped samples based on mean classification accuracy. Final model parameters were then obtained by fitting the models to the whole training dataset.

Heuristic Models

We chose QuickDASH item 9, QuickDASH item 11, and the EQ-5D-5L VAS as variables for the symptomatic improvement CHAID classifier. We chose QuickDASH item 1 and the EQ-5D-5L Mobility domain as variables for the functional improvement CHAID classifier. These variables were chosen based on Shapley values from the respective XGB algorithms, perceived ease of implementation, and clinical plausibility.

For each tree, the level of significance for splitting nodes and for merging predictor categories was set to $p < 0.05$. A minimum of 20 observations per node were required to undertake a split.

Supplementary Appendix: Supplementary Results

Missing Data

A missing data analysis (Supplementary Table 2 and Supplementary Table 3) suggested missing data were largely *missing at random*.[4] Compared to those with complete postoperative response sets, patients with missing follow-up responses were younger, had experienced symptoms for a longer time and had a higher BMI. Non-responders were more likely to be smokers and report depression. Responders were more likely to be currently employed, report high blood pressure and report osteoarthritis.

**Postoperative symptom scores**		**Not missing**	**Missing**	**P value**
**Age**		62.0 (14.6)	53.6 (14.8)	<0.001
**Gender**	Female	735 (57.6%)	540 (42.4%)	0.188
	Male	385 (60.9%)	247 (39.1%)	
**Symptom duration (months)**		35.0 (57.7)	45.4 (74.6)	0.001
**Previous injections**		4.7 (12.3)	3.6 (10.5)	0.136
**Previous operations**		4.1 (11.8)	3.5 (12.1)	0.487
**Preoperative splinting time (months)**		10.6 (14.3)	11.7 (16.0)	0.186
**Smoking status**	Non-smoker	967 (62.8%)	572 (37.2%)	<0.001
	Smoker	147 (40.9%)	212 (59.1%)	
**Heart disease**	No	970 (57.9%)	705 (42.1%)	0.118
	Yes	145 (63.6%)	83 (36.4%)	
**High blood pressure**	No	658 (55.1%)	536 (44.9%)	<0.001
	Yes	460 (64.9%)	249 (35.1%)	
**Lung disease**	No	1025 (58.6%)	723 (41.4%)	1.000
	Yes	86 (58.5%)	61 (41.5%)	
**Diabetes**	No	957 (59.9%)	641 (40.1%)	0.025
	Yes	161 (52.8%)	144 (47.2%)	
**Stomach ulcers**	No	1054 (58.8%)	737 (41.2%)	0.397
	Yes	58 (54.2%)	49 (45.8%)	
**Kidney disease**	No	1078 (58.6%)	761 (41.4%)	0.803
	Yes	36 (56.2%)	28 (43.8%)	
**Liver disease**	No	1101 (58.6%)	777 (41.4%)	0.862
	Yes	15 (62.5%)	9 (37.5%)	
**Anaemia**	No	1079 (58.9%)	753 (41.1%)	0.248
	Yes	32 (50.8%)	31 (49.2%)	
**Cancer**	No	1056 (58.4%)	752 (41.6%)	0.571
	Yes	54 (62.1%)	33 (37.9%)	
**Depression**	No	944 (62.4%)	568 (37.6%)	<0.001
	Yes	159 (42.3%)	217 (57.7%)	
**Osteoarthritis**	No	745 (56.2%)	580 (43.8%)	0.001
	Yes	360 (64.9%)	195 (35.1%)	

**Back pain**	No	725 (59.8%)	488 (40.2%)	0.223
	Yes	389 (56.8%)	296 (43.2%)	
**Rheumatoid arthritis**	No	1033 (58.3%)	738 (41.7%)	0.721
	Yes	66 (60.6%)	43 (39.4%)	
**Thyroid disease**	No	968 (58.7%)	680 (41.3%)	0.736
	Yes	133 (57.3%)	99 (42.7%)	
**Employment status**	Worker	1015 (61.2%)	643 (38.8%)	<0.001
	Unemployed	27 (34.6%)	51 (65.4%)	
	Long Term Sick / Disabled	60 (42.0%)	83 (58.0%)	
**BMI**		29.7 (6.4)	30.9 (7.7)	<0.001
**Diagnosis**	Carpal tunnel syndrome	1109 (58.5%)	786 (41.5%)	0.331
	Recurrent carpal tunnel syndrome	15 (71.4%)	6 (28.6%)	
**Undergoing surgery to dominant hand**	No	448 (61.1%)	285 (38.9%)	0.075
	Yes	640 (56.8%)	486 (43.2%)	

Supplementary Table 2. A comparison of patients who had complete and missing responses to the postoperative QuickDASH symptoms items. Continuous variables are presented as mean (standard deviation) and compared with Kruskal-Wallis tests. Categorical data are presented as counts (percentage) and compared with Chi squared tests. Co-morbidities were self-reported.

**Postoperative function scores**		**Not missing**	**Missing**	**p**
**Age**		61.7 (14.6)	54.5 (15.1)	<0.001
**Gender**	Female	701 (55.0%)	574 (45.0%)	0.119
	Male	372 (58.9%)	260 (41.1%)	
**Symptom duration (months)**		35.1 (56.8)	44.7 (74.7)	0.002
**Previous injections**		4.8 (12.5)	3.6 (10.3)	0.105
**Previous operations**		4.2 (12.0)	3.5 (11.8)	0.381
**Preoperative splinting time (months)**		10.7 (14.4)	11.5 (15.7)	0.332
**Smoking status**	Non-smoker	927 (60.2%)	612 (39.8%)	<0.001
	Smoker	140 (39.0%)	219 (61.0%)	
**Heart disease**	No	936 (55.9%)	739 (44.1%)	0.614
	Yes	132 (57.9%)	96 (42.1%)	
**High blood pressure**	No	636 (53.3%)	558 (46.7%)	0.001
	Yes	435 (61.4%)	274 (38.6%)	
**Lung disease**	No	991 (56.7%)	757 (43.3%)	0.160
	Yes	74 (50.3%)	73 (49.7%)	
**Diabetes**	No	917 (57.4%)	681 (42.6%)	0.031
	Yes	154 (50.5%)	151 (49.5%)	
**Stomach ulcers**	No	1013 (56.6%)	778 (43.4%)	0.257
	Yes	54 (50.5%)	53 (49.5%)	
**Kidney disease**	No	1036 (56.3%)	803 (43.7%)	0.381
	Yes	32 (50.0%)	32 (50.0%)	
**Liver disease**	No	1056 (56.2%)	822 (43.8%)	1.000
	Yes	14 (58.3%)	10 (41.7%)	
**Anaemia**	No	1031 (56.3%)	801 (43.7%)	0.815
	Yes	34 (54.0%)	29 (46.0%)	
**Cancer**	No	1013 (56.0%)	795 (44.0%)	0.564
	Yes	52 (59.8%)	35 (40.2%)	
**Depression**	No	904 (59.8%)	608 (40.2%)	<0.001
	Yes	155 (41.2%)	221 (58.8%)	
**Osteoarthritis**	No	716 (54.0%)	609 (46.0%)	0.001
	Yes	346 (62.3%)	209 (37.7%)	

**Back pain**	No	691 (57.0%)	522 (43.0%)	0.520
	Yes	379 (55.3%)	306 (44.7%)	
**Rheumatoid arthritis**	No	988 (55.8%)	783 (44.2%)	0.493
	Yes	65 (59.6%)	44 (40.4%)	
**Thyroid disease**	No	923 (56.0%)	725 (44.0%)	1.000
	Yes	130 (56.0%)	102 (44.0%)	
**Employment status**	Worker	977 (58.9%)	681 (41.1%)	<0.001
	Unemployed	24 (30.8%)	54 (69.2%)	
	Long Term Sick / Disabled	55 (38.5%)	88 (61.5%)	
**BMI**		29.6 (6.4)	30.9 (7.6)	<0.001
**Diagnosis**	Carpal tunnel syndrome	1062 (56.0%)	833 (44.0%)	0.233
	Recurrent carpal tunnel syndrome	15 (71.4%)	6 (28.6%)	
**Undergoing surgery to dominant hand**	No	431 (58.8%)	302 (41.2%)	0.066
	Yes	612 (54.4%)	514 (45.6%)	

Supplementary Table 3. A comparison of patients who had complete and missing responses to the postoperative QuickDASH function items (1-6). Continuous variables are presented as mean (standard deviation) and compared with Kruskal-Wallis tests. Categorical data are presented as counts (percentage) and compared with Chi squared tests. Co-morbidities were self-reported.

Class Balance

Prior to using the SMOTE, the symptom classifier training dataset contained 208 patients that did not improve and 611 that did improve. Following the SMOTE, the symptom classifier training dataset contained 624 patients in each class. In the function classifier training dataset, there were 398 patients that did not improve and 385 that did improve. We did not conduct the SMOTE in the function classifier training dataset.

Model Performance

Confusion matrices for each model are presented in Supplementary Table 4. In these matrices, a positive event (Yes) is an improvement following surgery that exceeds the minimal important change value for the relevant QuickDASH subscale. All predictions were made in the respective test datasets.

**Elastic Net (symptoms)**				**Elastic Net (function)**			
		Truth				Truth	
		No	Yes			No	Yes
Prediction	No	41	32	Prediction	No	101	42
	Yes	36	165		Yes	39	80

**XGB (symptoms)**				**XGB (function)**			
		Truth				Truth	
		No	Yes			No	Yes
Prediction	No	25	30	Prediction	No	92	23
	Yes	34	185		Yes	51	96

**SVM (symptoms)**				**SVM (function)**			
		Truth				Truth	
		No	Yes			No	Yes
Prediction	No	35	80	Prediction	No	97	35
	Yes	24	135		Yes	46	84

**ANN (symptoms)**				**ANN (function)**			
		Truth				Truth	
		No	Yes			No	Yes
Prediction	No	42	73	Prediction	No	83	30
	Yes	17	142		Yes	60	89

**KNN (symptoms)**				**KNN (function)**			
		Truth				Truth	
		No	Yes			No	Yes
Prediction	No	29	85	Prediction	No	91	39
	Yes	30	130		Yes	52	80

Supplementary Table 4. Confusion matrices for the functional and symptomatic improvement classifiers. EN: logistic regression with elastic net regularization; XGB: random forest built through extreme gradient boosting; SVM: support vector machine; ANN: artificial neural network; KNN: K nearest neighbors.

[illegible]odel Explanations

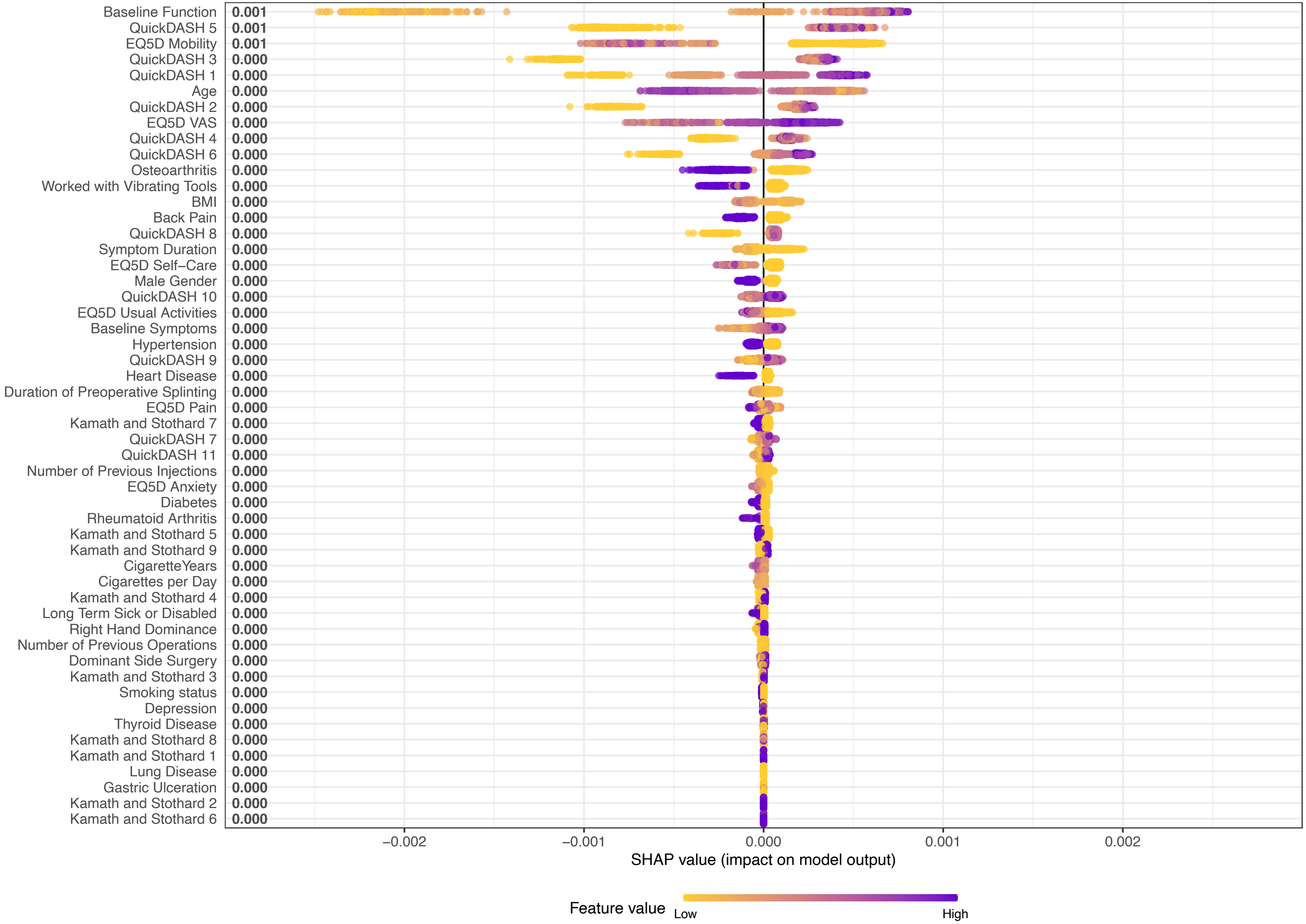

[illegible]upplementary figure 1. Shapley additive explanations for the functional improvement classifier (XGB) predictions on the test data.

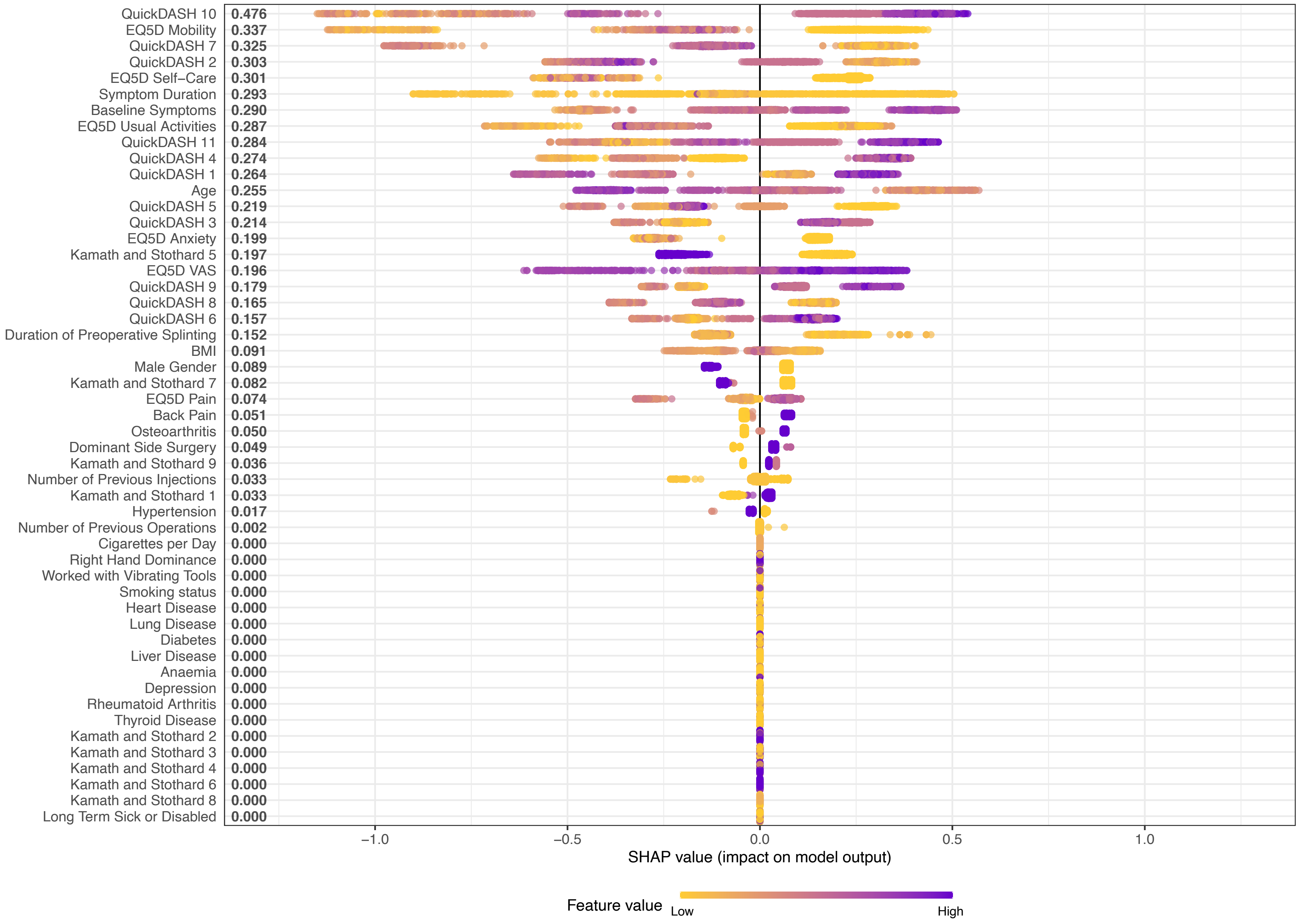

Supplementary figure 2. Shapley additive explanations for the symptomatic classifier (XGB) predictions on the test data.

References

1. Chawla NV, Bowyer KW, Hall LO, Kegelmeyer WP. SMOTE: Synthetic Minority Over-sampling Technique. *J Artif Intell Res*. 2002;16:321-357. doi:10.1613/jair.953

2. Kuhn M, Wickham H, RStudio. Package “tidymodels.” CRAN. Published 2021. Accessed August 27, 2021. https://cran.r-project.org/web/packages/tidymodels/index.html

3. Kuhn M, RStudio. Package “tune.” CRAN. Published 2021. Accessed August 27, 2021. https://cran.r-project.org/web/packages/tune/tune.pdf

4. Mack C, Su Z, Westreich D. *Managing Missing Data in Patient Registries: Addendum to Registries for Evaluating Patient Outcomes: A User’s Guide, Third Edition*. Agency for Healthcare Research and Quality (US); 2018. Accessed August 27, 2021. http://www.ncbi.nlm.nih.gov/books/NBK493611/
